# Künstliche Intelligenz in der internistischen Versorgung: Von der Theorie zum praktischen Einsatz in Praxen und Kliniken

**DOI:** 10.1007/s00108-023-01604-z

**Published:** 2023-10-17

**Authors:** Jens Eckstein

**Affiliations:** 1 Klinik für Innere Medizin, Universitätsspital Basel, Basel, Schweiz; 2 Innovationsmanagement, Universitätsspital Basel, Hebelstr. 10, 4031 Basel, Schweiz

**Keywords:** Künstliche Intelligenz/Akzeptanz, Beziehung zwischen Patient und Gesundheitsfachperson, Entscheidungsunterstützungssysteme, Erklärbare künstliche Intelligenz, Digitale Transformation, Artificial intelligence/acceptance, Professional-patient relationship, Decision support systems, Artificial intelligence, explainable, Digital transformation

## Abstract

Die Integration von Technologien der künstlichen Intelligenz (KI) hat das Potenzial, sowohl die Effizienz als auch die Qualität der medizinischen Versorgung zu verbessern. In verschiedenen Fachbereichen der Inneren Medizin haben KI-Anwendungen bereits ihren festen Platz, während sich die Anwendungen in anderen Bereichen noch in unterschiedlichen Phasen der Entwicklung befinden. Ein wichtiger zu beleuchtender Aspekt sind die Auswirkungen der KI auf die Interaktion zwischen Patienten und Gesundheitsfachpersonen. Ein weiterer Faktor ist die Nachvollziehbarkeit der Funktionsweise involvierter KI-basierter Algorithmen. Neben notwendigen vertrauensbildenden Maßnahmen ist eine Integrierbarkeit der Technologie in bestehende Systeme anzustreben, um eine entsprechende Akzeptanz und breite Verfügbarkeit zu erreichen und die Mitarbeiter:innen auf administrativer Ebene zu entlasten.

## Abstract (en)

The integration of artificial intelligence (AI) technologies has the potential to improve both the efficiency and the quality of medical care. Applications of AI have already become established in various specialized fields in internal medicine, whereas in other fields the applications are still in various phases of development. An aspect that is important to elucidate is the effects of AI on the interaction between patients and healthcare personnel. A further factor is the comprehensibility of the mode of functioning of the AI-based algorithms involved. In addition to the necessary confidence-building measures, an integration of the technology into existing systems must be strived for to achieve an appropriate acceptance and widespread availability and to relieve pressure on the personnel at the administrative level.

Die fortschreitende Entwicklung von künstlicher Intelligenz (KI) hat einen erheblichen Einfluss auf verschiedene Bereiche unseres täglichen Lebens, so auch im Gesundheitswesen. Die Integration von KI-Technologien hat das Potenzial, sowohl die Effizienz als auch die Qualität der medizinischen Versorgung zu verbessern.

Die Anwendung von KI in der Inneren Medizin ist aktuell je nach Fachbereich noch sehr unterschiedlich ausgeprägt

Im Spektrum der Versorgung internistischer Patient:innen werden zunehmend Anwendungsmöglichkeiten für KI-basierte Systeme identifiziert (Abb. 1) und zum Teil bereits erfolgreich um- und eingesetzt. Im vorliegenden Beitrag werden verschiedene Beispiele der KI-Anwendung im Gesundheitswesen untersucht, darunter diagnostische, therapeutische und administrative Anwendungen. Darüber hinaus werden die Auswirkungen der KI auf die Interaktion zwischen Patienten und Gesundheitsfachpersonen diskutiert.

**Figure 1:**
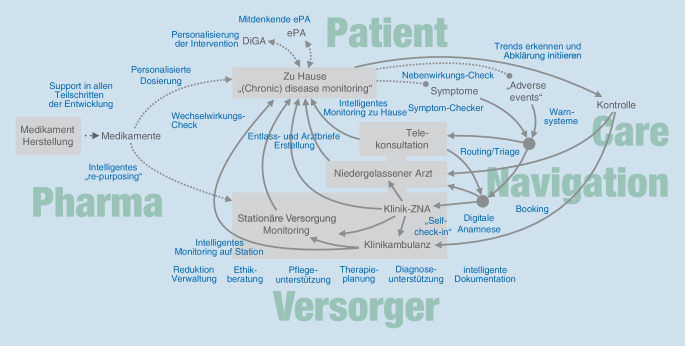

## Vertrauen und Nachvollziehbarkeit

Besondere Herausforderungen bei der Anwendung von KI-basierten Algorithmen in der Medizin sind zum einen die hohen Anforderungen an die fachliche Verlässlichkeit der ausgegebenen Resultate im Falle von Entscheidungsunterstützungsprogrammen und zum anderen das Bedürfnis nach Nachvollziehbarkeit des Entscheidungswegs. Diese Konstellation führt dazu, dass KI-Anwendungen, bei denen die zugrunde liegenden Quelldaten den Anwender:innen für eine eigene Beurteilung zur Verfügung stehen, schneller Vertrauen schaffen als solche, bei denen ein möglicherweise plausibles, aber nicht überprüfbares Resultat ausgegeben wird [1, 2]. Für beides sei ein Beispiel genannt.

Überprüfbarkeit der Quelldaten einer KI-Anwendung fördert die Akzeptanz

Eine frühe, inzwischen fest in den Leitlinien verankerte Anwendung ist die Nutzung von Photoplethysmographie(PPG)-Signalen, die mittels Translumineszenz der Haut beispielsweise mit Smartphone-Kameras oder Wearables (mobile Sensoren) erzeugt werden. Dabei wird die pulssynchron wechselnde Signalintensität des durch die Haut reflektierten Lichts aufgezeichnet und mittels eines KI-basierten Algorithmus analysiert. So kann beispielsweise Vorhofflimmern ausgeschlossen oder als wahrscheinlich identifiziert werden. Die Technologie wird von internationalen Fachgesellschaften für das Vorhofflimmerscreening empfohlen. Seit 2020 wird zudem die Diagnose von Vorhofflimmern mittels mobiler Elektrokardiogramme (EKG) und KI-gestützter Analyse akzeptiert [3]. Wichtig ist, dass die zuständigen Ärzt:innen bei diesen Anwendungen neben den KI-basierten Befunden immer noch die Originalaufzeichnung verifizieren können. Dieser Umstand hat die vergleichsweise rasche Einführung und hohe Akzeptanz befördert.

In einem anderen Beispiel entwickelten Forscher der Mayo-Klinik ein KI-basiertes Programm, das basierend auf einem normalen Sinusrhythmus-EKG vorherzusagen vermag, ob die Person in der Zukunft Vorhofflimmern bekommen wird. Für die zuständigen Ärzt:innen besteht aber keine Möglichkeit zu überprüfen, auf welchen Faktoren dieser Befund basiert [4]. Derartige Anwendungen verlangen ein hohes Maß an Vertrauen in den KI-basierten Befund; entsprechend ist zu erwarten, dass die Akzeptanz langsamer wächst.

## Anwendungsbeispiele

### Kardiologie.

Das oben beschriebene PPG-Signal mobiler Sensoren erlaubt neben dem Vorhofflimmerscreening noch eine Vielzahl weiterer KI-Anwendungen. Bereits als Medizinprodukt erhältlich ist ein Device für die Langzeitblutdruckmessung. Der zugrunde liegende Algorithmus ist nicht im Detail bekannt, macht sich aber die blutdruckabhängige Veränderung der Pulswellenmorphologie zunutze [5].

### Endokrinologie.

In der Diabetesbehandlung können KI-gesteuerte Systeme kontinuierlich Glukosewerte überwachen und Insulinverabreichungen anpassen, um den Blutzuckerspiegel in einem gesunden Bereich zu halten. Diese Technologie, auch als „künstliches Pankreas“ bekannt, kann die Lebensqualität von Menschen mit Diabetes erheblich verbessern. Durch die Analyse von Blutzuckerdaten und anderen relevanten Parametern kann die KI eine optimale Insulindosis berechnen und die automatische Verabreichung auslösen [6, 7].

### Rheumatologie.

In der Rheumatologie kann KI bei Diagnosestellung und Monitoring von Erkrankungen wie rheumatoider Arthritis unterstützen. Durch die Analyse von Labordaten, Bildern und klinischen Informationen kann KI Ärzten helfen, den Krankheitsverlauf besser zu verstehen und individuellere Behandlungspläne zu entwickeln. Dies kann dazu beitragen, die Lebensqualität der Patienten zu verbessern und Schübe der Erkrankung frühzeitig zu erkennen (siehe auch der ergänzende Kurzbeitrag in der vorliegenden Ausgabe).

### Nephrologie.

KI kann in der Nephrologie bei der Überwachung von Patienten mit akutem Nierenversagen und chronischer Nierenerkrankung eine wichtige Rolle spielen. Durch die Analyse von Laborergebnissen, klinischen Daten und Bildern kann KI Frühwarnzeichen von Nierenproblemen identifizieren und Ärzte bei der Entscheidungsfindung unterstützen. Dies ermöglicht eine rechtzeitige Intervention, um den Krankheitsverlauf zu verlangsamen oder zu stabilisieren [8].

### Gastroenterologie.

In der Gastroenterologie kann KI bei der Erkennung von Darmpolypen und anderen Darmveränderungen helfen, indem eine KI-gesteuerte Bildanalyse verdächtige Läsionen frühzeitig erkennt. Die Früherkennung von Polypen kann die Wirksamkeit der Darmkrebsvorsorge erhöhen und die Behandlungschancen verbessern [9].

### Intensivmedizin.

KI kann in der Intensivmedizin eine entscheidende Rolle bei der Vorhersage einer Sepsis spielen. Durch die kontinuierliche Überwachung von Vitalparametern, Laborwerten und Patientendaten kann KI frühe Anzeichen einer sich entwickelnden Sepsis erkennen. Dies ermöglicht dann eine frühzeitige Intervention und eine Optimierung der Behandlung [10].

### Infektiologie.

In der Infektiologie kann KI bei der Erkennung und Behandlung von Infektionen eine wichtige Rolle spielen. Durch die Analyse von klinischen Daten, Bildern, Labordaten und epidemiologischen Informationen kann KI die Wahrscheinlichkeit einer Infektion bewerten und Behandlungsempfehlungen geben. Dies ist besonders relevant in Situationen von Ausbrüchen oder Pandemien [11].

Ein weiterer, für das Monitoring internistischer Patient:innen interessanter Parameter ist die Körperkerntemperatur. Gerade während der Coronavirus-disease-2019(COVID-19)-Pandemie war Fieber ein zentraler Indikator für eine behandlungsbedürftige Infektion [12]. Hier besteht eine gewisse Unsicherheit durch die Angabe von Wearable-Herstellern, die Körpertemperatur zu messen. De facto wird meist die lokale Hauttemperatur am Handgelenk ermittelt und mit Algorithmen, oft unter Berücksichtigung weiterer Faktoren wie Außen- und Gerätetemperatur, eine mutmaßliche Körperkerntemperatur berechnet. Die Werte mögen grundsätzlich korrekt ermittelt sein, spiegeln aber nicht zuverlässig die in der Medizin gebräuchliche Körperkerntemperatur wider. Zumal bei akut kranken Patient:innen physiologisch eine Zentralisierung mit nachfolgender Absenkung der peripheren Körpertemperatur einsetzt. Hier wäre ein KI-Algorithmus gefragt, der basierend auf einer multifaktoriellen Analyse diesen Umstand entweder zu kompensieren vermag oder zumindest ausweist, dass die aktuell ermittelten Werte nicht die reale Körperkerntemperatur darstellen. Eine vielversprechende Alternative bieten Hersteller von Wärmeflusssensoren, welche die lokale Wärmeabstrahlung registrieren und mittels proprietärer Algorithmen daraus die Körperkerntemperatur errechnen [13].

### Psychiatrie.

Die aus der Pulswelle abgeleitete Herzfrequenzvariabilität kann als unspezifischer, aber sensitiver Parameter beim Monitoring von Patient:innen mit Depressionen und Burn-out verwendet werden [14, 15]. Entsprechende Anwendungen halten allmählich Einzug in die klinische Routine. Ziel dieser Entwicklung ist, neben einer Therapiekontrolle in der Akutphase, eine intermittierende langfristige Begleitung der Patient:innen. So kann zum einen den Patient:innen ein positives Feedback gegeben werden, zum anderen können potenzielle Rezidive möglichst früh erkannt und behandelt werden.

### Bildgebende Diagnostik.

Eine der derzeit wirkmächtigsten Anwendungen von KI im Gesundheitswesen liegt im Bereich der Bilddiagnostik. KI-gestützte Algorithmen können medizinische Bilder wie Röntgenaufnahmen, computertomographische Scans und Magnetresonanztomogramme analysieren, um Anomalien und Erkrankungen frühzeitig zu erkennen. Dies kann die Genauigkeit von Diagnosen verbessern und zu einer schnelleren und genaueren Behandlung führen. Beispiele sind KI-Systeme zur Erkennung von malignen Läsionen oder zur Diagnose von ophthalmologischen Erkrankungen wie der diabetischen Retinopathie [16, 17].

Auch hier bestehen die Prozesse in einer Voranalyse durch die KI und einer finalen Befundung durch die fachärztlichen Kolleg:innen. Neben der potenziellen Qualitätsverbesserung durch vermehrte Detektion potenziell pathologischer Befunde dient diese Art der Anwendung von KI der Entlastung der befundenden Kolleg:innen durch die KI-basierte „Vorbefundung“ [18].

### KI-unterstützte Sonographie mit Handhelds.

Die zunehmend verfügbaren mobilen Ultraschallgeräte haben das Potenzial, die Routinediagnostik in der Inneren Medizin nachhaltig zu verändern. Dank technologischer Neuerungen sind diese Geräte nicht nur deutlich günstiger und robuster, sie verfügen unter anderem auch über eine KI-basierte Auswertungssoftware, welche die Fachperson entsprechend unterstützt [19]. Zu klären sind hier für den europäischen Markt noch Fragen des Datenschutzes und der Datenspeicherung. Es ist aber gut vorstellbar, dass die KI-gestützte Handheld-Sonographie das traditionelle Stethoskop in Zukunft ersetzen wird.

## Zusammenspiel zwischen Gesundheitsfachperson, künstlicher Intelligenz und Patient:in

Die Integration von KI im Gesundheitswesen wirft wichtige Fragen zur Patienten-Gesundheitsfachpersonen-Interaktion auf. Einerseits kann KI den Zugang zu medizinischem Wissen und Informationen erleichtern. KI-gestützte Plattformen können Patienten mit relevanten Informationen über ihre Gesundheit versorgen und ihnen helfen, selbstständig Entscheidungen zu treffen. Zum Beispiel können KI-Chatbots Patienten über Symptome und mögliche Behandlungsoptionen aufklären [20]. Andererseits besteht die Gefahr, dass der menschliche Aspekt der medizinischen Versorgung vernachlässigt wird. Die emotionale Unterstützung und das zwischenmenschliche Verständnis, die von menschlichen Gesundheitsfachpersonen geboten werden, können durch KI nicht vollständig ersetzt werden. Eine ausgewogene Integration von KI und menschlicher Interaktion ist von entscheidender Bedeutung [21]. Daraus leitet sich die Vision einer zukünftigen „Zusammenarbeit“ von Gesundheitsfachperson, KI und Patient:in ab.

## Visualisierung und Zuordnung

Ein weiteres wichtiges Arbeitsfeld ist die Darstellung, Interpretation und Bewertung der zunehmend großen Datenmengen in einer intuitiv interpretierbaren Form. Auch hier findet sich wieder der Aspekt der Nachvollziehbarkeit und somit des Vertrauens wieder. Ein klassischer Langzeit-EKG-Befund zeigt sowohl eine Analyse als auch die Rohdaten der entsprechenden interpretierten Sequenz auf.

Im Hinblick auf die große Anzahl von Patient:innen mit eigenen Sensoren wird es auch immer wichtiger zu verstehen, woher die Messwerte stammen. Auch wenn die von den Patienten verwendeten Sensoren nicht zertifiziert sind, können sie mit einem entsprechenden Label, das auf die Herkunft und potenziell eingeschränkte Validität hinweist, einen Mehrwert bieten. An dieser Stelle sei der Vergleich mit einer guten Anamnese erlaubt. Auch hier geht es darum, möglichst alle Informationen der Patient:innen zu erhalten und sie dann in einem weiteren Schritt abzuwägen und zu gewichten.

Ebenso wie in der bisherigen Medizin abgewogen werden musste, welches die geeignete Diagnostik oder Therapie für unsere Patient:innen ist, wird man auch beim Einsatz neuer Technologien eine passende Auswahl treffen müssen: beginnend bei der Akzeptanz für mobile Sensoren, welche die Patient:innen zuverlässig und korrekt tragen oder einsetzen müssen, bis zur Auswahl der korrekten Algorithmen, welche für die jeweilige Fragestellung getestet sein müssen. Dies bedeutet im Umkehrschluss für die Ärzteschaft, dass sie für den Umgang mit KI-basierten Algorithmen verstehen muss, was der „intended use“ bei deren Entwicklung war. Ein einfaches Beispiel wäre die Tatsache, dass der KI-basierte Algorithmus eines Fitnessarmbands, der zum Zählen von Schritten für Athleten entwickelt wurde, die Schritte von Menschen mit Rollator kaum korrekt bestimmen kann, weil sie während des Gehens ihre Hände ruhig an den Griffen des Hilfsmittels haben.

## Administrative Entlastung

Durch die Anwendung von KI besteht ein großes Potenzial, das Gesundheitssystem bei administrativen Aufgaben zu entlasten. Bereits verfügbar sind KI-Algorithmen, welche die Plausibilität von Abrechnungen prüfen. In Entwicklung befindlich ist die halbautomatische Erstellung von Arztbriefen, die aktuell noch einen relevanten Teil der ärztlichen Arbeitsbelastung ausmachen. Durch die KI-basierte „Vorbereitung“ solcher Dokumente, die dann für eine finale Version noch gegengelesen und gegebenenfalls korrigiert werden müssen, sollte das Personal von repetitiven Aufgaben entlastet werden und somit wieder mehr Zeit für die direkte Interaktion mit Patient:innen haben [22].

## Integration in medizinische Systeme

Damit Patient:innen von den skizzierten Vorteilen profitieren können, muss ein Ziel der weiteren Entwicklung sein, dass derartige Systeme tief in die medizinische Versorgung integriert und die erhobenen Daten mittels KI-basierter Algorithmen automatisch analysiert werden können, um die ärztliche Intervention auf die medizinisch notwendigen Situationen zu reduzieren. Grundlage hierfür wären neben einer entsprechenden technischen Infrastruktur zur sicheren direkten Übermittlung der Daten auch geeignete medizinische Sensoren, die spezifisch für derartige Zwecke entwickelt und getestet wurden. Mit einem System aus geeigneten mobilen Sensoren, stabiler und sicherer Infrastruktur sowie automatischer, KI-basierter Analyse und Bewertung ließen sich gleichzeitig Sicherheit und Qualität der Patient:innenversorgung steigern und die Ressourcen im Gesundheitswesen entlasten.

Derartige Systeme sollen explizit nicht nur in der Klinik, sondern auch bei Patient:innen zu Hause zum Einsatz kommen, um bei Bedarf ein kontinuierliches ambulantes Monitoring zu ermöglichen. Diesem Trend folgend ist derzeit eine Zunahme des Angebots von Monitoringsystemen für den häuslichen Kontext zu verzeichnen. Diese firmieren häufig unter dem Begriff „hospital at home“ oder „home monitoring“. Ziel der Bemühungen muss ein validiertes System für unterschiedliche Einsatzorte sein, das medizinischen Qualitätsansprüchen genügt und Kliniken, Kolleg:innen in eigener Praxis, Forschenden und Patient:innen in gleichem Maße zur Verfügung steht [23].

Um die damit einhergehende Skalierung bezüglich der Menge an Messdaten bewältigen zu können, sind wiederum KI-basierte Algorithmen im Hintergrund unabdingbar. Diese Systeme müssen eine ständige Analyse der erhobenen Parameter sicherstellen und vordefinierte Schritte auslösen, sobald beispielsweise eine ärztliche Intervention notwendig wird. Hier ist hervorzuheben, dass dies bei Systemen, die einen einzelnen Parameter wie etwa den Herzrhythmus überwachen, einfacher ist als bei Systemen, die beispielsweise aus einem Zusammenspiel von Körperkerntemperatur, Herzfrequenz, Atemfrequenz, Sauerstoffsättigung und Aktivität den Verlauf einer Lungenentzündung erkennen sollen. Um bei durchschnittlichen internistischen Patient:innen Anwendung zu finden, ist dieser Grad an Komplexität jedoch Voraussetzung. Dementsprechend zielt die Forschung und Entwicklung darauf ab, Systeme zu entwickeln und im klinischen Einsatz zu testen, die auch bei komplexen Patient:innen sicher (keine falsch-negativen Meldungen) und effizient sind (möglichst wenig falsch-positive Meldungen). Basierend auf den Erfahrungen mit sogenannten „early warning scores“ (EWS) ist zu erwarten, dass sich dadurch die Versorgung der Patient:innen weiter verbessert, weil Verschlechterungen des Gesundheitszustands frühzeitiger erkannt und entsprechende Maßnahmen früher eingeleitet werden können [23].

## Fazit für die Praxis

- Die Integration von künstlicher Intelligenz (KI) im Gesundheitswesen bietet immense Chancen, die Patientenversorgung zu transformieren.
- Durch die Automatisierung administrativer Aufgaben, die Verbesserung von Diagnosen und die Personalisierung von Therapien können KI-Technologien die medizinische Praxis verbessern und effizienter machen.
- Dabei müssen wir die Auswirkungen auf die menschliche Interaktion, den Datenschutz und die Transparenz sorgfältig abwägen.
- KI sollte der Unterstützung von Gesundheitsfachpersonen dienen, um die Qualität der Versorgung zu steigern und gleichzeitig die menschliche Empathie und Fachkenntnis zu bewahren.
